# Staged male genital reconstruction with a local flap and free oral graft: a case report and literature review

**DOI:** 10.1186/s12894-019-0537-6

**Published:** 2019-10-29

**Authors:** Da-chao Zheng, Min-kai Xie, Shi-bo Fu, Jian-hua Guo, Wen-ji Li, Hai-jun Yao, Zhong Wang

**Affiliations:** Department of Urology, Shanghai 9th People’s Hospital, Shanghai JiaoTong University School of Medicine, NO.369, ZhiZaoJu Road, HuangPu District, Shanghai, 200011 China

**Keywords:** Genital reconstruction, Penile urethra defect, Local flap, Free oral graft

## Abstract

**Background:**

Male genital skin loss is a common disease in urology. However, male genital skin loss accompanying a penile urethra defect is rarely reported. Herein, we describe a novel surgical technique using a composite local flap and oral mucosal graft to reconstruct the penis, which may provide a new solution for patients with similar conditions.

**Case presentation:**

A 36-year-old male with a penile urethra defect and a large area of genital skin loss required urethral reconstruction. The meatus had descended to the penoscrotal junction. This procedure was divided into three stages. The first stage of the surgery involved burying the nude penile shaft beneath the skin of the left anteromedial thigh for coverage of the skin defect. The second stage consisted of releasing the penis and expanding the size of the urethral plate for further urethroplasty. The third stage consisted of reconstruction of the anterior urethra 6 months later. Postoperatively, the patient reported satisfactory voiding. The maximal flow rate (MFR) was 22.2 ml/s with no postvoiding residual urine at the 24-month follow-up visit. No edema, infection, hemorrhage, or cicatricial retraction were observed. The patient’s erectile function was satisfactory, and his international index of erectile function-5 score (IIEF-5 score) was 23 at the 24-month follow-up visit. Additionally, the presence of nocturnal penile tumescence demonstrated that he had normal erectile function.

**Conclusions:**

This procedure is an effective surgical option for men with complete foreskin and penile urethra defects. It could also be extended as a treatment strategy when composite local or pedicle transposition flaps and free grafts are needed for specific patients.

## Background

Male genital skin loss following severe infections such as Fournier’s gangrene or injuries is a common condition in urology. Reconstruction with local pedicled penile flaps. Scrotal flaps, split-thickness skin grafting (STSG) and/or pedicle flaps is an effective technique when the urethra is completely preserved [1, 2]. The use of several materials such as genital or extragenital skin or mucosa has also been described to repair pure urethral defects [3, 4]. However, reports describing a solution for male genital skin loss accompanying a penile urethra defect are rare.

This paper aimed to report a unique case of complete genital skin loss and a penile urethral defect due to a severe traffic accident. We designed a novel staged procedure and successfully treated this patient.

## Case presentation

### Patient information and clinical findings

A 36-year-old male survived a severe traffic accident but lost his right leg, bilateral testicles and a large area of genital skin, including the foreskin and scrotal skin. Additionally, the penile urethra was damaged, resulting in a urethral defect 6 cm in length, and the meatus had descended to the penoscrotal junction.

### Therapeutic focus and assessment

**Fig. 1:**
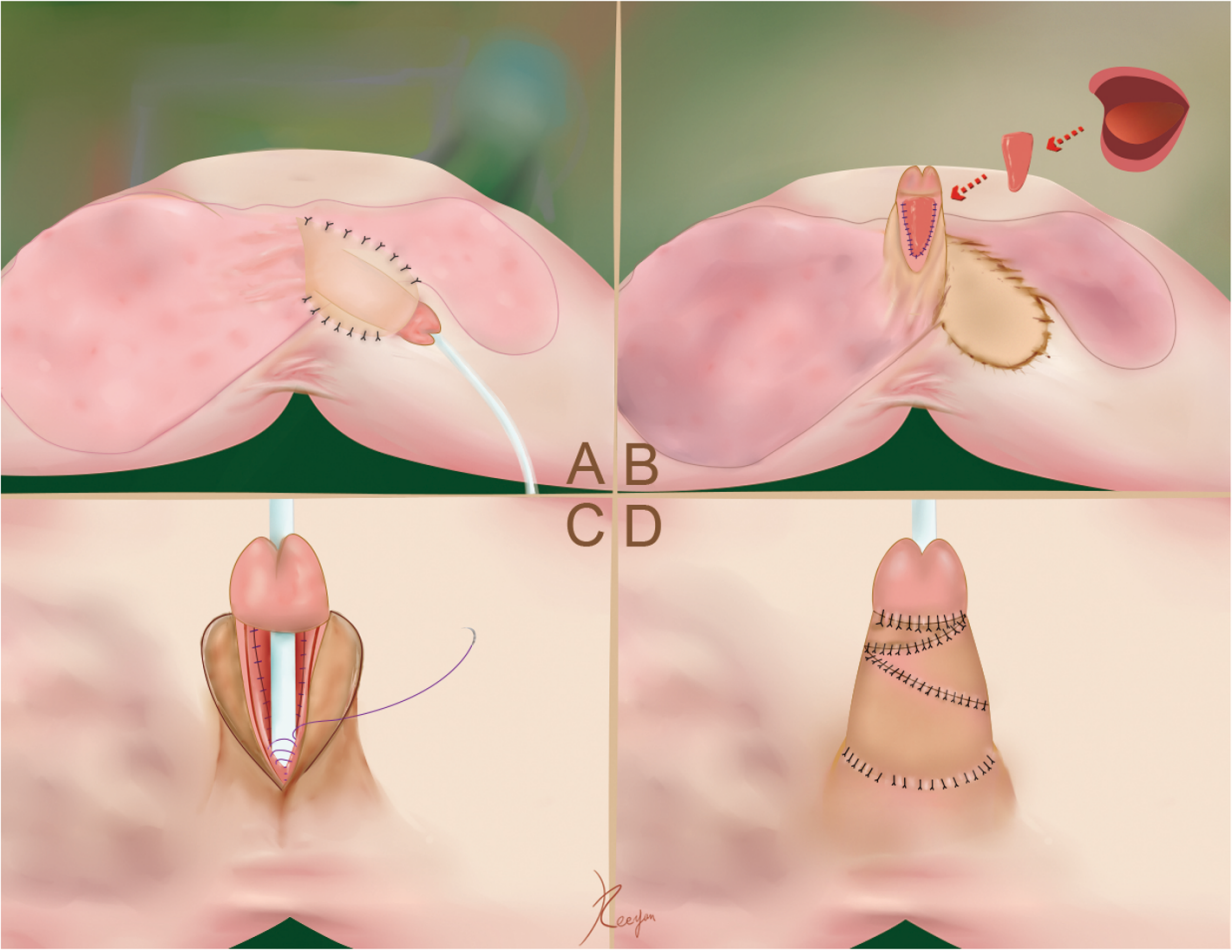
**a**. Penile protection and flap preparation: The nude penile shaft was buried beneath the skin of the left anteromedial thigh; **b**. Penile release and urethral plate expansion: The penis was released, and a free lingual mucosal graft was harvested to expand the size of the urethral plate; **c**. Penile and urethral reconstruction: A standard Thiersch-Duplay technique was performed on the preset neourethral plate. **d**. The preset flap was utilized for tension free coverage of the corpus cavernosum and neourethra

The patient strongly desired to perform standing urination; therefore, we performed a three-stage procedure to repair his penis. In the first stage, skin saving measures and coverage of the skin loss were the most important treatments. Free skin grafts and pedicle flaps were transposed to repair the skin defects. The nude penile shaft was buried beneath the skin of the left anteromedial thigh (Fig. 1a).

Twelve months later, the patient underwent the second-stage procedure for penile release and urethral plate expansion (Fig. 1b). In this stage, the penis was released from the left thigh and was fully covered with skin and subcutaneous fat. The size of the penile dorsal skin was designed to be large enough to wrap the corpus cavernosum and residual urethral plate. Considering the shrinkage of the residual urethral plate, a lingual mucosal graft [5] was harvested to expand the urethral plate. The neourethral plate was designed to be as large as possible due to the expected 20% shrinkage of the oral graft [6]. A protective tie-over dressing was placed to reduce the chance of hematoma collecting under the graft. No focal graft contracture occurred requiring an additional operation to patch the graft, and no donor site complications were observed at the follow-up evaluation (Additional file 1).

At 6 months after the second-stage surgery, a third stage was performed for urethroplasty. In this stage, the patient underwent a urethroplasty procedure utilizing the preset flap and the neourethral plate with a standard Thiersch-Duplay technique (Fig. 1c) (Additional file 2). The neourethra was tabularized with 2-layer running subepithelial 6–0 Vicryl sutures. Adequate dissection of the new foreskin, excision of a part of the subcutaneous adipose tissue and Z-plasty led to tension free coverage of the corpus cavernosum and neourethra (Fig. 1d) (Additional file 3). The 12-Fr catheter was removed on postoperative day (POD) 21, and no fistula was observed after the urethral catheter was removed.

At the 2-year follow-up evaluation after the repair, the patient voided satisfactorily (Additional files 4 and 5). The maximal flow rates (MFRs) were 27.8 ml/s, 23.3 ml/s, and 22.2 ml/s with no postvoiding residual urine after the third-stage operation and at the 12-month and 24-month follow-up evaluations, respectively. No edema, infection, hemorrhage, or cicatricial retraction were observed. The patient reported penile erectile function and the ability to perform intercourse without penile curvature. A mild reduction of penile hardness and sensitivity were reported during the 2-year follow-up visit. However, his IIEF-5 scores were 22 and 23 at the 12-month and 24-month follow-up evaluations, respectively, as demonstrated by nocturnal penile tumescence (NPT) during postoperative year 2.

## Discussion and conclusions

Genital skin loss is usually caused by Fournier’s gangrene, trauma, lymphedema, tumors and other diseases [2, 7, 8]. Reconstruction of the urethra and penile or scrotal skin defects with good functional and cosmetic results presents challenges for surgeons. The selection of proper techniques depends on the size of the defect, condition of the local tissue and status of the wound [9].

STSG is a viable treatment option for patients without urethral injuries. The free skin graft can be harvested rapidly to cover large defects [10] and provide a resurfacing solution for the genital region and perineum. However, for this patient, STSG was not feasible. The main disadvantages were as follows. First, the free grafts commonly undergo contraction when they survive. According to our preoperative design, the new foreskin was prepared for coverage of the cavernous bodies and neourethra during the urethroplasty stage. This disadvantages would cause the new foreskin to have an insufficient size and lead to a poor expansive ability for wrapping the neourethra in the final stage. Second, the lack of subcutaneous tissue and abundant blood is another disadvantage for the survived free skin grafts that leads to a poor healing capacity and a high probability of fistula or necrosis in the urethroplasty stage.

To overcome the disadvantages of STSG, fasciocutaneous or musculocutaneous flaps, including the scrotal skin flap, gracilis myocutaneous flap [11], pedicle anterolateral thigh flap [12], and anteromedial thigh fasciocutaneous flap [13], were used, which produced excellent cosmetic outcomes. These flaps had reliable circulation and provided sufficient size, good flexibility and subcutaneous tissues for the penile shaft. However, there were two contradictions. One was that our patient needed a rapid resurfacing for his penis, but these flap techniques would require a long time. The other was the need for STSG for skin defects, which required healthy skin for a donor site. Therefore, burying the nude penile shaft beneath the skin of the left anteromedial thigh was adopted. This technique had been mentioned in a classic textbook describing the treatment of pure penile skin loss using scrotal skin [14]. The skin of the left anteromedial thigh was the nearest healthy skin that could provide similar characteristics to other flaps that were transferred from other donor sites. The greatest advantage of our method was the ability to prefabricate chimeric flaps.

Various techniques for urethral reconstruction have been described [4, 15, 16]. Flaps and grafts, and even the appendix and intestinal segments, have been used as alternative techniques [17–21], and most of these techniques were staged procedures. Yazar [9] and colleagues reported a one-stage technique to repair a complex penile defect with composite anterolateral thigh and vascularized fascia lata flaps. In that case, the vascularized fascia lata flap was utilized to repair the lateral and ventral semicircular wall defect of the urethra. They chose this technique because the patient lacked a well-vascularized recipient bed for the surviving free graft. In this situation, the vascularized fascia lata flap could provide reliable circulation for urethroplasty. In contrast, our patient had a vascularized recipient bed for grafts, and the grafts could be easily harvested. Thus, transforming the penis into a penoscrotal hypospadias for an easier operation was a better choice. Moreover, we had no time to harvest such a composite anterolateral thigh and vascularized fascia lata flap in the first stage. Our method was not as creative as Yazar’s, but it was much safer for the repair of the urethral defect. If the graft developed local contracture and necrosis, an additional operation could be performed to patch the graft. Furthermore, an additional operation could be performed to harvest a vascularized fascia lata flap from the other leg when Yazar’s patient experienced operative failure, but we had no room for failure because our patient had only one leg.

In the second stage, the lingual mucosa was utilized to expand the urethral plate for urethroplasty. Oral mucosal grafts have been demonstrated to be an effective technique for urethroplasty [22, 23]. Buccal mucosa grafting is another choice, and the selection depends on the surgeon’s preference. We did not choose an onlay graft technique or a tubed graft technique because of the high breakdown rates, which may be related to the lack of an adequately vascularized graft bed [24]. The dorsal inlay grafting technique that we selected could provide a large, hairless and well-vascularized neourethral plate for urethroplasty. This was the one of the reasons why we performed three operations.

In a standard hypospadias repair, fistula is a major complication, and meatal stenosis, strictures, infection, and chordee are other common complications [25]. Barrier flap coverage with scrotal dartos flaps or tunica vaginalis flaps is a routine procedure in the treatment of hypospadias and decreases the incidence of fistulas [26, 27]. This patient had lost his genital skin and testicles. Fortunately, the preset flap with reliable circulation and a large amount of subcutaneous tissue provided a good wound healing, and no fistula was observed. Stricture is another major complication of urethroplasty. For this patient, a semi-circular anastomosis was performed to prevent stricture. The satisfactory wound healing and anti-infective ability of the preset flap played an important role in the prevention of stricture. Finally, multiple Z-patterns were designed to reduce the tension of the wounds and the risk of chordee.

In this case, penile erection and subsequent sexual intercourse were preserved postoperatively. Although the final outcomes including the function and cosmetic appearance were satisfactory, the patient still had a binding sensation. This may be associated with a large area of scar tissue on the abdominal, perineal and penile skin. The patient also experienced a mild reduction of penile hardness and sensitivity after the operations, although rigiscan testing indicated that he still had satisfactory erectile function. We suspected that the lack of a penile urethra and foreskin were the contributing factors.

Our result showed that this staged procedure was a simple, effective and safe technique. Furthermore, this approach is also practicable for surgeons who have not mastered complicated flap techniques. Although this procedure was time consuming, we still recommend it for cases with complete genital skin loss and penile urethra defects. Moreover, this staged technique can be improved as a treatment strategy by using proper composite local or pedicle transferred flaps and free grafts for repair of complete genital skin and urethra defects. However, longer follow-up and additional cases are needed to further evaluate the continued use of this technique.

## Supplementary information

**Additional file 1.** Preoperative status: The penis was released, and a free lingual mucosal graft was preset as new urethral plate for the urethraplasty.

**Additional file 2.** Urethroplasty: A standard Thiersch-Duplay technique was performed on the preset neourethral plate.

**Additional file 3.** Postoperative status: The figure showed the cosmetic outcome of operation.

**Additional file 4.** Postoperative status 2 yrs.: The postoperative cosmetic appearance of penis after 2 years.

**Additional file 5.** Urination:The video demonstrate a normal voiding without any complications.

## Data Availability

The datasets analyzed during the current study are available from the corresponding author on reasonable request.

## Abbreviations

IIEF-5: International index of erectile function-5 score
MFR: Maximal flow rate
NPT: Nocturnal penile tumescence
POD: Postoperative day
STSG: Split-thickness skin grafting
