# Supplementary figures and images for: Staged male genital reconstruction with a local flap and free oral graft: a case report and literature review
Source: BMC Urol. 2019 Oct 29;19:104. doi: 10.1186/s12894-019-0537-6 (PMC6819581; doi:10.1186/s12894-019-0537-6)

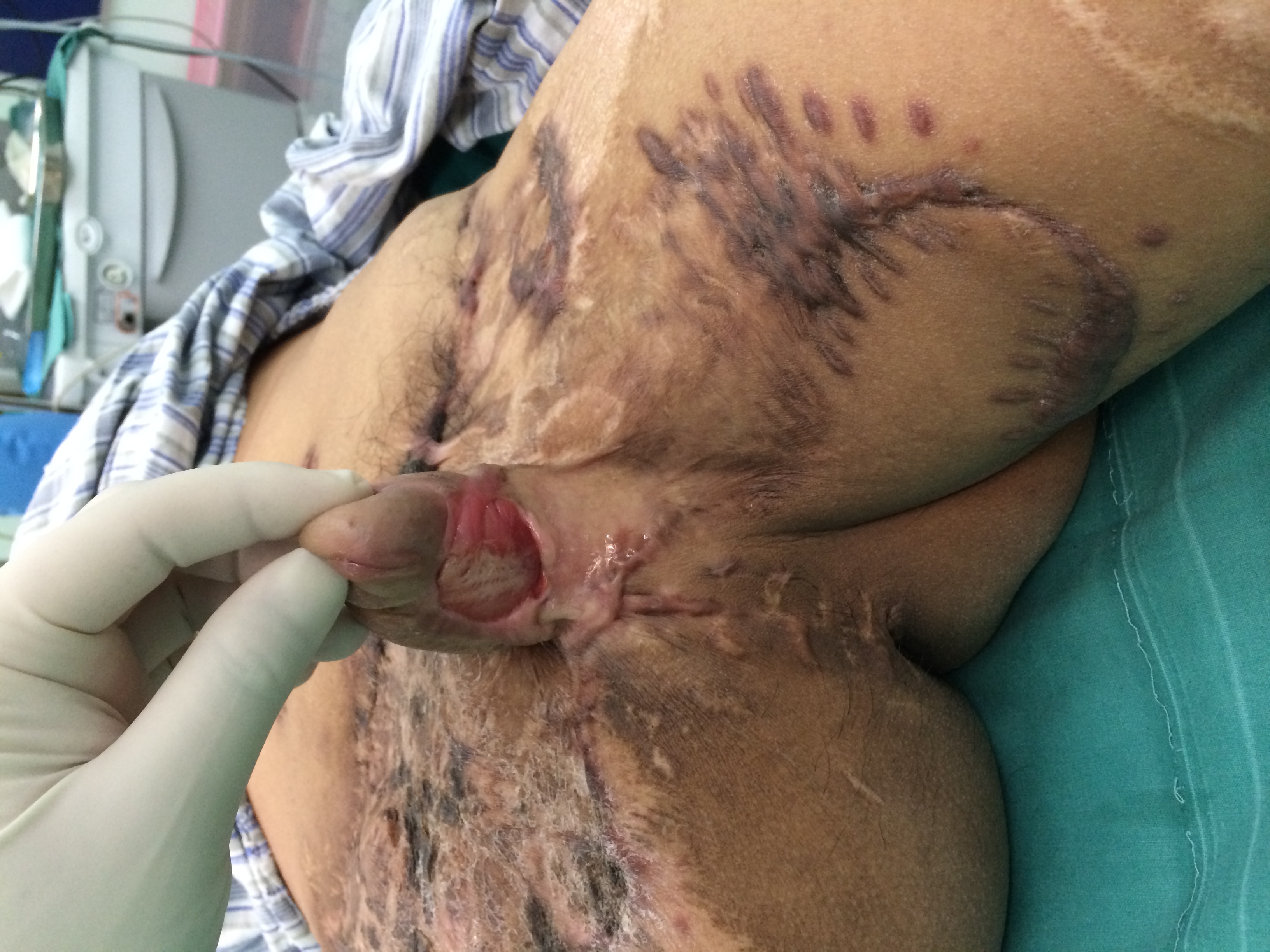

Supplement: Supplementary file 1 — Additional file 1. Preoperative status: The penis was released, and a free lingual mucosal graft was preset as new urethral plate for the urethraplasty. [file 12894_2019_537_MOESM1_ESM.jpg]

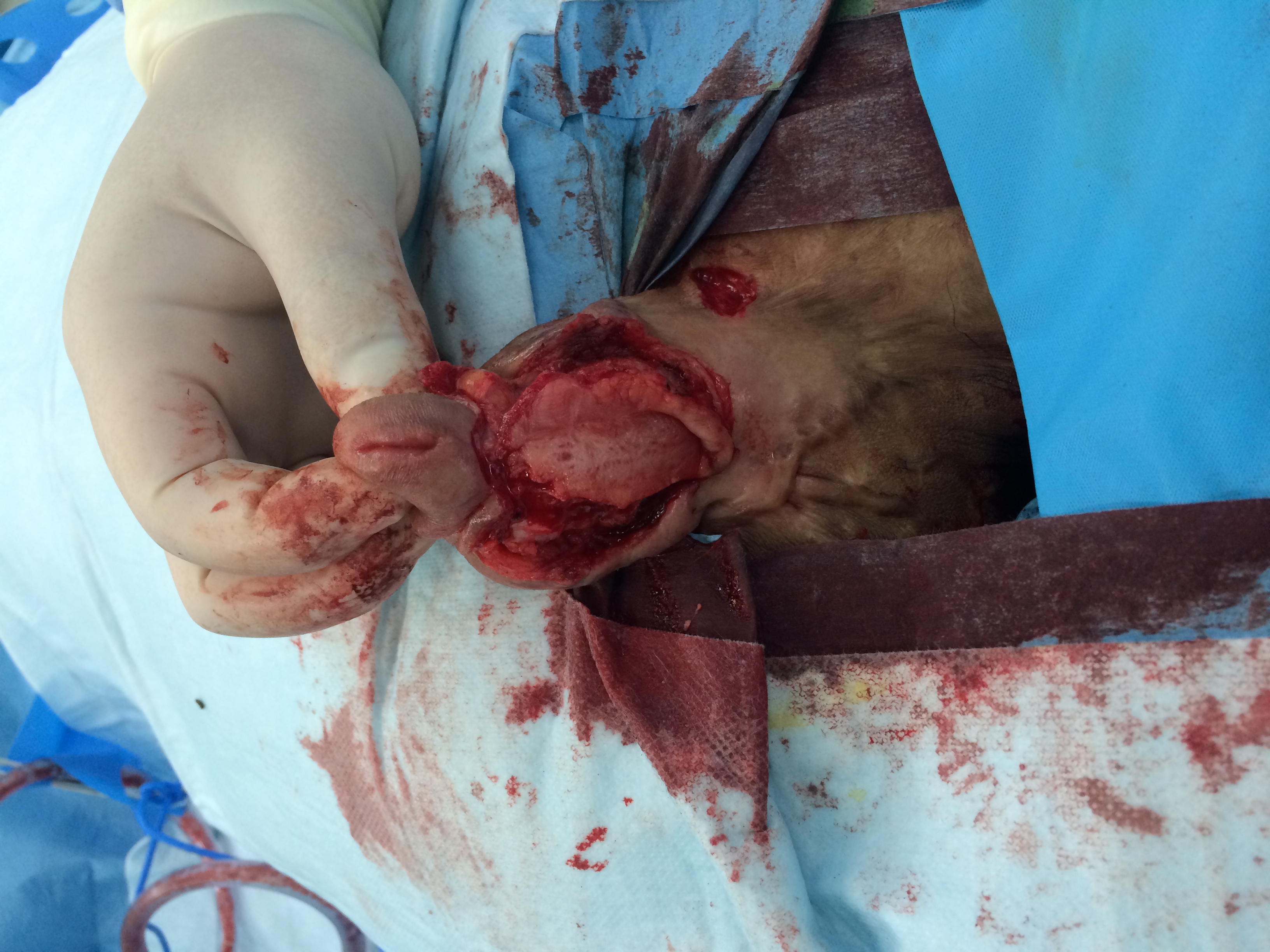

Supplement: Supplementary file 2 — Additional file 2. Urethroplasty: A standard Thiersch-Duplay technique was performed on the preset neourethral plate. [file 12894_2019_537_MOESM2_ESM.jpg]

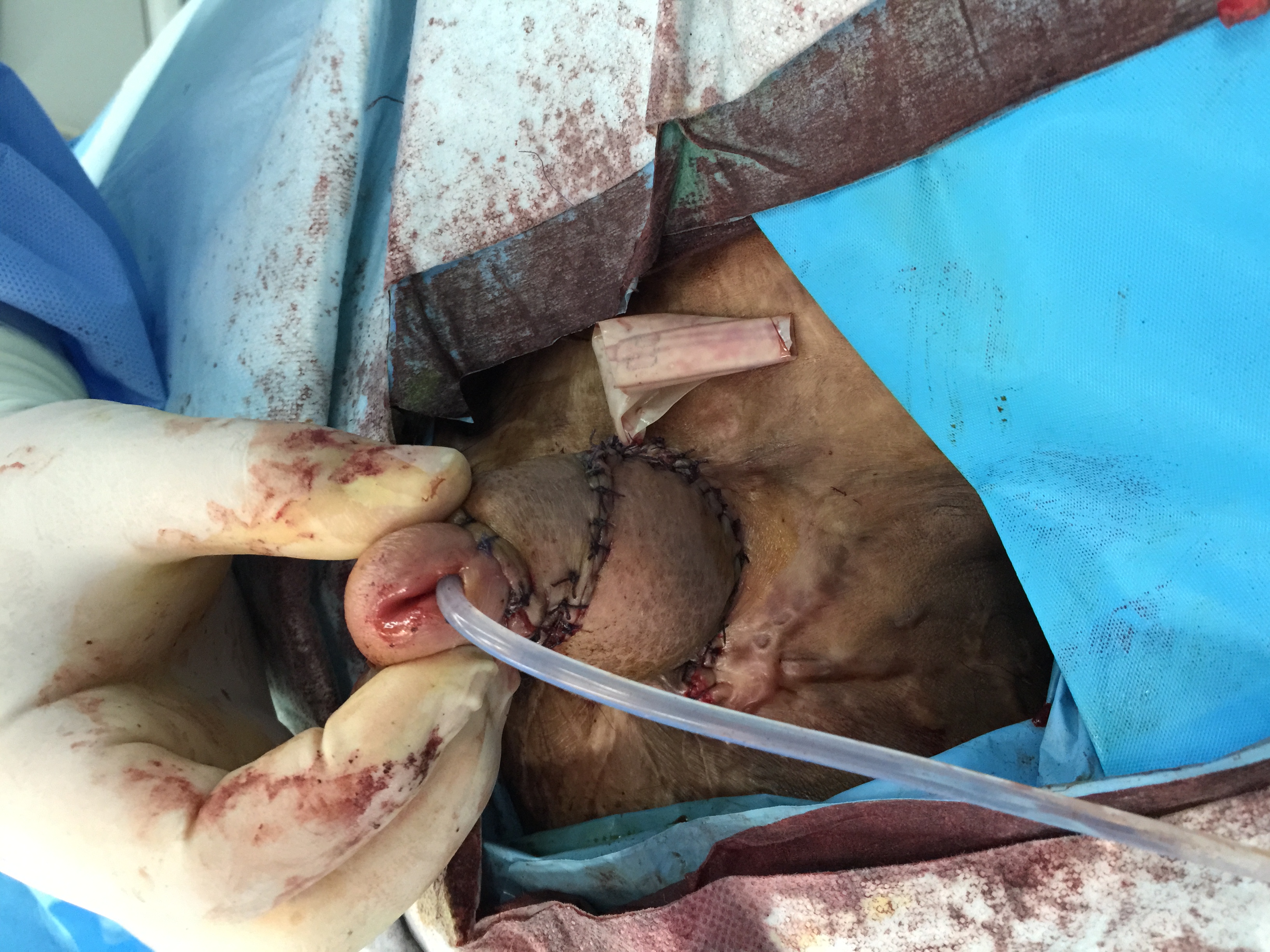

Supplement: Supplementary file 3 — Additional file 3. Postoperative status: The figure showed the cosmetic outcome of operation. [file 12894_2019_537_MOESM3_ESM.jpg]

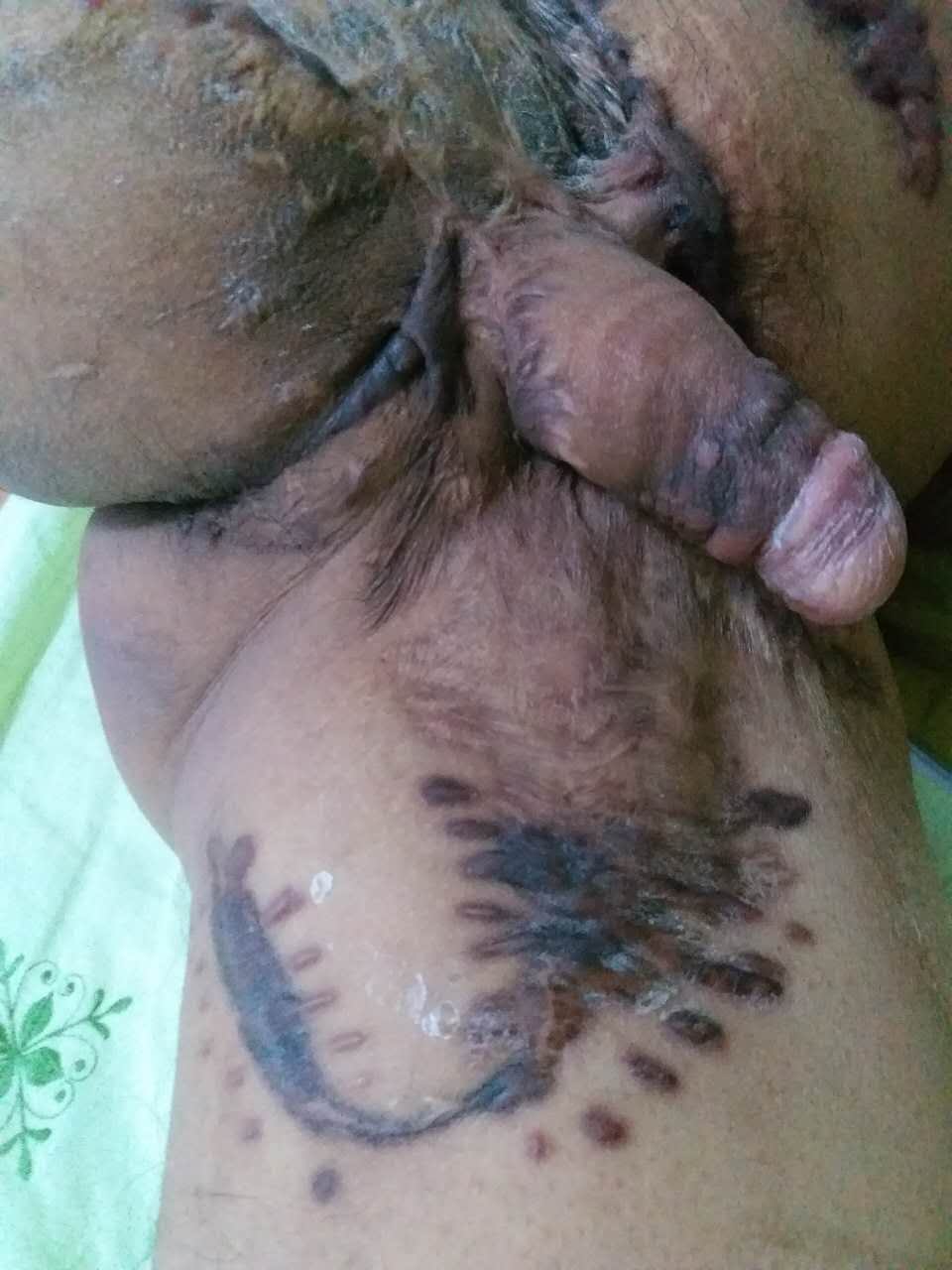

Supplement: Supplementary file 4 — Additional file 4. Postoperative status 2 yrs.: The postoperative cosmetic appearance of penis after 2 years. [file 12894_2019_537_MOESM4_ESM.jpg]
